# A real-world pharmacovigilance study of adverse drug reactions associated with lecanemab and aducanumab based on WHO-VigiAccess and FAERS databases

**DOI:** 10.3389/fphar.2025.1561020

**Published:** 2025-04-01

**Authors:** Haoxiang Hu, Yunhan Zhao, Jiesheng Mao, Jianghai He, Yihan Zhang, Hongyu Ye, Xiaokai Yang

**Affiliations:** Department of Neurology, Postgraduate training base Alliance of Wenzhou Medical University (Wenzhou People’s Hospital), Wenzhou, China

**Keywords:** lecanemab, aducanumab, alzheimer’s disease (AD), VigiAccess, FAERS

## Abstract

**Background:**

Lecanemab and Aducanumab are two novel anti-amyloid beta (Aβ) therapies for Alzheimer’s disease (AD) that have shown promise in slowing cognitive decline. However, their safety profiles remain unclear due to limited real-world evidence. This study aims to analyze and compare adverse drug reactions (ADRs) of these drugs using data from the WHO-VigiAccess and FAERS databases.

**Methods:**

A retrospective analysis was conducted using ADR data from the VigiAccess and FAERS databases, focusing on System Organ Class (SOC) and Preferred Term (PT) classifications. Descriptive statistics and reporting odds ratio (ROR) analysis were employed to evaluate and compare ADR profiles.

**Results:**

Lecanemab and Aducanumab exhibited distinct ADRs. Results from both the VigiAccess and FAERS databases indicated that the most SOC associated with both drugs was nervous system disorders (34.7% in VigiAccess, 36.8% in FAERS). Further multivariable logistic regression analysis revealed that Aducanumab was associated with a higher risk of nervous system disorders (OR = 4.72, 95% CI: 3.53–6.39, P < 0.001). Among the reported AEs, headache was the most frequently reported for Lecanemab (9.4% in VigiAccess, 8.96% in FAERS), while Aducanumab was primarily associated with amyloid-related imaging abnormalities (ARIA) (19.1% in VigiAccess, 23.58% in FAERS). In the blood and lymphatic systems, Anemia was observed in both drugs. However, thrombocyto-penia was more prevalent in Lecanemab, while platelet dysfunction and myelosuppression were more frequently observed in Aducanumab. Additionally, hospitalization and mortality rates were higher for Aducanumab compared to Lecanemab.

**Conclusion:**

This study compared the ADRs of Lecanemab and Aducanumab, revealing that ARIA was the most common AE for both drugs. However, Lecanemab showed a lower risk of ARIA, cerebral hemorrhage, and severe events. These findings emphasize the need for further clinical research to clarify the long-term safety and efficacy of both drugs.

## Introduction

Alzheimer’s disease (AD) is the most prevalent form of dementia, affecting over 50 million individuals globally. This number is projected to triple, reaching approximately 152 million cases worldwide (Liu et al., 2024). AD imposes substantial healthcare costs on society, places a heavy burden on families, and markedly diminishes the quality of life for affected individuals (Lane et al., 2018). Traditional therapeutic approaches primarily focus on inhibiting acetylcholinesterase and modulating the neuronal function of N-methyl-D-aspartate (NMDA) receptors. However, these treatments yield limited efficacy, providing only temporary symptom relief without altering the fundamental disease progression (Long and Holtzman, 2019; Breijyeh and Karaman, 2020; Marasco, 2020).

The current amyloid hypothesis posits that the abnormal accumulation of β-amyloid (Aβ) protein in the brain serves as a critical pathogenic event, triggering complex cascade reactions that ultimately lead to tau pathology, neurodegeneration, and cognitive decline (Hardy and Selkoe, 2002). This hypothesis provides a significant theoretical basis for the development of novel pharmacological strategies to treat AD (Panza et al., 2019). Evidence suggests that anti-Aβ therapies may slow disease progression (van Dyck et al., 2023). Aducanumab, a monoclonal antibody, was the first drug to receive accelerated approval from the U.S. Food and Drug Administration (FDA) for the treatment of AD, based on its potential clinical benefit (Budd Haeberlein et al., 2022). Reports indicate that its most common adverse reactions (ADRs) are amyloid-related imaging abnormalities (ARIA), which are associated with cerebral edema (ARIA-E) and cerebral hemorrhage (ARIA-H) (Herring et al., 2021). Aducanumab reports showed 128 peaks in 2022 (44.1%) and 2023 (39.0%). Aducanumab was discontinued in Feb-May 2024 (Alzheimer’s Association, 2024). Lecanemab received full FDA approval in July 2023, with its therapeutic efficacy supported by clinical trials. Studies demonstrate that Lecanemab consistently reduces Aβ deposition and tau aggregation in the brain (Swanson et al., 2021; van Dyck et al., 2023). Its primary ADR is infusion-related reactions, generally mild to moderate in severity. The incidence of ARIA-E was reported to be 12.6% compared to placebo, with most cases resolving between 4 and 16 weeks post-onset (Moutinho, 2022).

Clinical trials of Lecanemab and Aducanumab have both demonstrated a certain degree of improvement in the progression of AD. A critical aspect of their clinical application lies in comprehensively understanding their unique safety profiles. However, as Lecanemab and Aducanumab have only recently been introduced into clinical practice, the current understanding and in-depth investigation of their associated adverse events (AEs) are still in their infancy and remain highly controversial (Budd Haeberlein et al., 2022; Reardon, 2023; Mahase, 2024). Moreover, clinical trials are often constrained by factors such as limited sample sizes, incomplete trial completion rates, and the restricted number of identified ADRs. These limitations make it challenging to fully elucidate the AE characteristics of these drugs. Therefore, this study uniquely leverages data from the WHO-VigiAccess and FAERS databases, aiming to characterize the AE profiles closely associated with Lecanemab and Aducanumab, identify potential novel AE signals, and compare the differences in AE profiles between the two drugs. Through these novel findings, we aim to provide clinicians, patients, and regulatory agencies with a more comprehensive understanding of the safety profiles of Lecanemab and Aducanumab. This will enable a balanced assessment of their risks and benefits, guide personalized treatment decisions, and promote safer and more effective therapeutic practices. Additionally, this study establishes a crucial foundation for future pharmacovigilance efforts and subsequent safety research.

## Materials and methods

### Data sources

**FIGURE 1:**
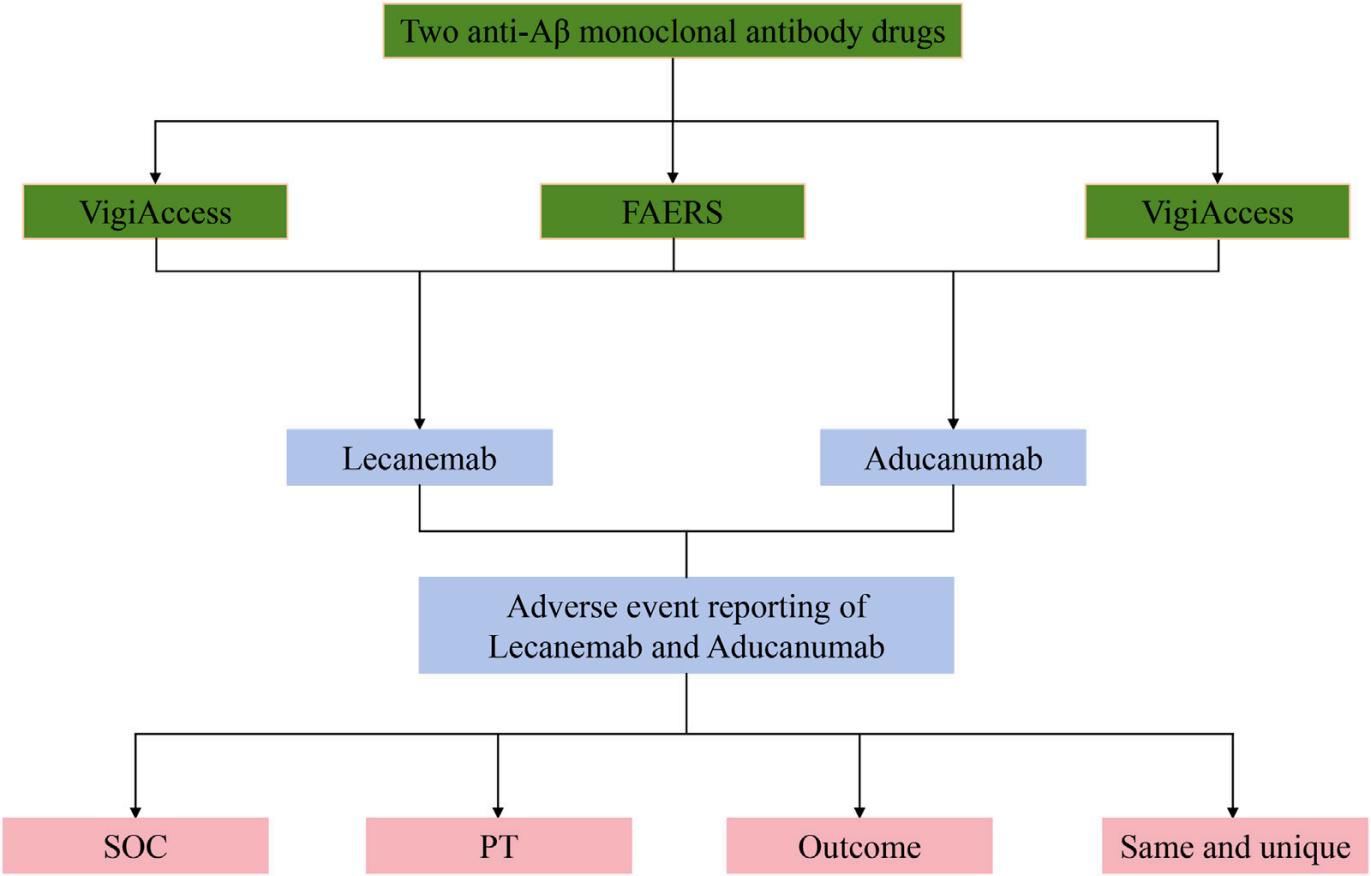
The flowchart of this study. Footnotes: SOC, System Organ Class; PT, Preferred Term.

All AEs reported after the use of Lecanemab and Aducanumab were extracted from the WHO-VigiAccess database (as of December 2024) and the FAERS database (as of the third quarter of 2024). WHO-VigiAccess is a free-access portal of the PIDM database that retrieves drug safety reports received by the Uppsala Monitoring Centre (UMC). This database aggregates AE reports from multiple countries and regions, offering a comprehensive global perspective on drug safety information. It provides ADR data that includes information on age, gender, continent, and reporting years (Huang et al., 2024). FAERS is a publicly available drug safety reporting database that collects AE reports from the United States and other countries, offering a robust collection of case data. FAERS database is updated on a quarterly basis, and since the fourth quarter’s adverse drug reaction data for 2024 has not yet been released, our retrospective analysis extends up to the third quarter of 2024. Together, these databases encompass a broad spectrum of drug users and serve as essential repositories of real-world AE occurrences, providing a rich source of data for this study. Based on the retrieved data, this study conducted an objective analysis of AEs associated with Lecanemab and Aducanumab. Adverse events were encoded using Preferred Terms (PTs) from the Medical Dictionary for Regulatory Activities (MedDRA) (Brown, 2004). PTs represent medical concepts associated with symptoms and disease diagnoses. The terminology in MedDRA is derived from various sources, including the World Health Organization Adverse Reaction Terminology (WHO-ART). The classification of AEs was further standardized using the System Organ Class (SOC) categorization framework (Tieu and Breder, 2018). Donanemab was only approved by the FDA in the third quarter of 2024, and ADR data for Donanemab had not been updated as of the third quarter of 2024; therefore, data mining and analysis of Donanemab was not performed in this study (Kang, 2024). A detailed workflow of this study is presented in Figure 1.

### Statistical analysis

This study employed a retrospective quantitative analysis approach, incorporating statistical descriptive analysis and data visualization. The ADR reporting rate for each drug was defined as the ratio of reported ADR symptoms to the total number of ADR reports. Descriptive variables were summarized using frequencies and percentages for categorical data. At the SOC and PT levels, a commonly used disproportionality analysis method, the reporting odds ratio (ROR), was applied to assess differences between Lecanemab and Aducanumab (Bao et al., 2024). A higher ROR value indicates a stronger AE signal, signifying a more significant statistical association between the target drug and the specific AE (Yang et al., 2024). To strengthen the study and avoid overrepresentation of ADRs, univariate and multivariate logistic regression analyses were further conducted in the FAERS database. However, due to the lack of essential demographic information and access restrictions in the VigiAccess database, we were unable to perform logistic regression analysis in this database, which is a limitation inherent to it. All analyses were conducted using R (version 4.4).

ROR=acbd

$$95\%CI=e^{\ln\left(ROR\right)\pm 1.96\sqrt{\frac{1}{a}+\frac{1}{b}+\frac{1}{c}+\frac{1}{d}}}$$

## Results

### Descriptive analysis of the two drugs in VigiAccess

The VigiAccess database reported 1,122 ADR reports for Lecanemab and 468 ADR reports for Aducanumab. Among the 1,590 ADR reports involving these two drugs, after excluding cases with unknown gender, Lecanemab-related ADRs were reported in 430 males (38.3%) and 588 females (52.4%). In comparison, Aducanumab-related ADRs were reported in 208 males (44.4%) and 232 females (49.6%). It’s worth noting that ADRs for Lecanemab were predominantly reported in the age group of 65 years or older, accounting for 65.8%. Similarly, ADRs for Aducanumab were also concentrated in the population aged 65 years or older, representing 52.7%. However, it should be noted that a significant proportion (over 40%) of Aducanumab’s reports did not provide the patient’s age information. Geographically, AE reports for both drugs primarily originated from the Americas (98.9% for Lecanemab and 96.2% for Aducanumab), while the proportion of AE reports from Asia, Africa, and Oceania was relatively low. Additionally, the number of AE reports for Lecanemab has significantly increased since 2023, while the number of AE reports for Aducanumab has notably risen since 2022 (Table 1).

**TABLE 1:** Baseline characteristics of Lecanemab and Aducanumab distribution in VigiAccess database.

Description	Lecanemab	Aducanumab
Number of events	1,122	468
Gender		
Female	588 (52.4%)	232 (49.6%)
Male	430 (38.3%)	208 (44.4%)
Unknown	104 (9.3%)	28 (6.0%)
Age (years)		
Under 45 years	1 (0.1%)	0 (0.0%)
45–64 years	111 (9.9%)	31 (6.6%)
65–74 years	321 (28.6%)	105 (22.4%)
≥75 years	417 (37.2%)	142 (30.3%)
Unknown	272 (24.2%)	190 (40.6%)
Region		
Americas	1,110 (98.9%)	450 (96.2%)
Asia	5 (0.4%)	1 (0.2%)
Europe	5 (0.4%)	12 (2.6%)
Oceania	2 (0.2%)	4 (0.9%)
Africa	0 (0.0%)	1 (0.2%)
Reporting year		
2024	983 (87.6%)	73 (15.6%)
2023	132 (11.8%)	160 (34.2%)
2022	6 (0.5%)	206 (44.0%)
2021	1 (0.1%)	10 (2.1%)
2018	0 (0.0%)	1 (0.2%)
2016	0 (0.0%)	18 (3.8%)

### Descriptive analysis of two drugs in FAERS

**FIGURE 2:**
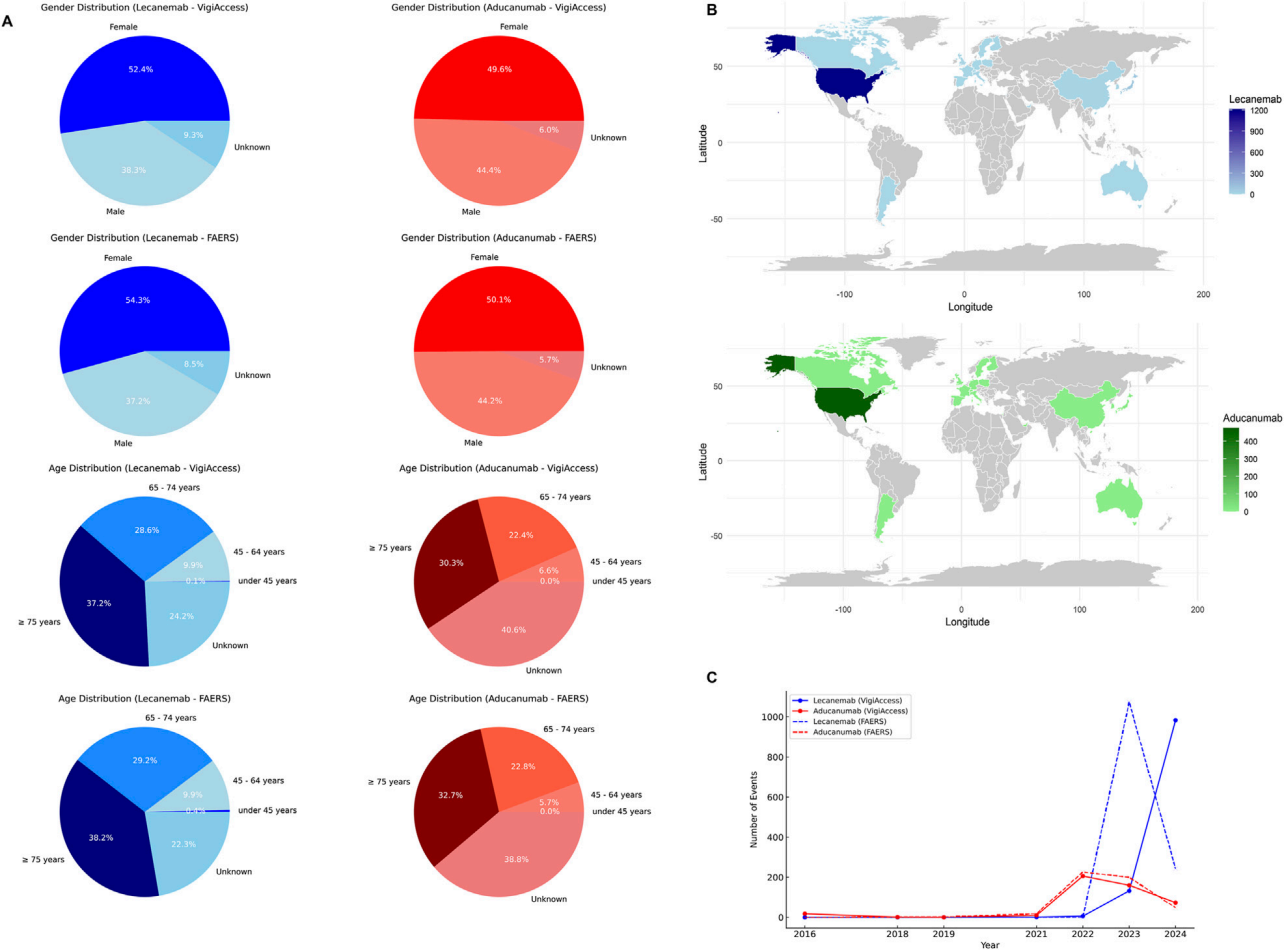
Baseline Characteristics of Lecanemab and Aducanumab Distribution. Footnotes: **(A)** The distribution of Lecanemab and Aducanumab by gender, and age group in VigiAccess and FAERS databases; **(B)** The distribution of Lecanemab and Aducanumab by country in FAERS database; **(C)** The distribution of Lecanemab and Aducanumab by reporting year in VigiAccess and FAERS databases.

The FAERS database reported 1,317 ADRs for Lecanemab and 513 for Aducanumab. After excluding reports of unknown gender, ADRs for Lecanemab were lower in males (37.2%) than females (54.3%). Similarly, Aducanumab reports followed the same trend, with male ADRs accounting for 44.2% and female ADRs for 50.1%. Consistent with the VigiAccess findings, Lecanemab ADRs were predominantly reported in individuals aged 65 years or older (67.4%). Likewise, Aducanumab ADRs were concentrated in the same age group (55.5%). It should be noted that both drugs had a proportion of reports lacking information on patient gender and age. In terms of geographic distribution, the majority of Lecanemab ADR reports originated from the United States (92.1%), with minimal contributions from other regions. Similarly, Aducanumab ADR reports were also mainly from the United States (92.6%), with other regions contributing minimally. Regarding the temporal distribution, the number of Lecanemab ADR reports peaked significantly in 2023 (81.5%), while Aducanumab reports showed peaks in 2022 (44.1%) and 2023 (39.0%) (Table 2). Figure 2 illustrates the data distribution from VigiAccess and FAERS in detail.

**TABLE 2:** Baseline characteristics of Lecanemab and Aducanumab distribution in FAERS database.

Description	Lecanemab	Aducanumab
Number of events	1,317	513
Gender		
Female	715 (54.3%)	257 (50.1%)
Male	490 (37.2%)	227 (44.2%)
Unknown	112 (8.5%)	29 (5.7%)
Age (years)		
under 45 years	6 (0.4%)	0 (0.0%)
45–64 years	130 (9.9%)	29 (5.7%)
65–74 years	384 (29.2%)	117 (22.8%)
≥75 years	503 (38.2%)	168 (32.7%)
Unknown	294 (22.3%)	199 (38.8%)
Country		
Argentina	2 (0.2%)	0 (0%)
Australia	2 (0.2%)	0 (0%)
Canada	1 (0.1%)	5 (1.0%)
China	11 (0.8%)	0 (0%)
France	4 (0.3%)	4 (0.8%)
Israel	1 (0.1%)	1 (0.2%)
Italy	3 (0.2%)	2 (0.4%)
Japan	69 (5.2%)	10 (1.9%)
Korea, South	3 (0.2%)	0 (0%)
Spain	2 (0.2%)	3 (0.6%)
Sweden	1 (0.1%)	1 (0.2%)
Switzerland	2 (0.2%)	4 (0.8%)
United Kingdom	3 (0.2%)	1 (0.2%)
United States	1,213 (92.1%)	475 (92.6%)
United Arab Emirates	0 (0%)	1 (0.2%)
Finland	0 (0%)	3 (0.6%)
Germany	0 (0%)	2 (0.4%)
Poland	0 (0%)	1 (0.2%)
Reporting year		
2024	243 (18.5%)	48 (9.4%)
2023	1,074 (81.5%)	200 (39.0%)
2022	0 (0%)	226 (44.1%)
2021	0 (0%)	18 (3.5%)
2019	0 (0%)	2 (0.4%)
2018	0 (0%)	1 (0.2%)
2016	0 (0%)	18 (3.5%)

### SOC distribution of the two drugs

**FIGURE 3:**
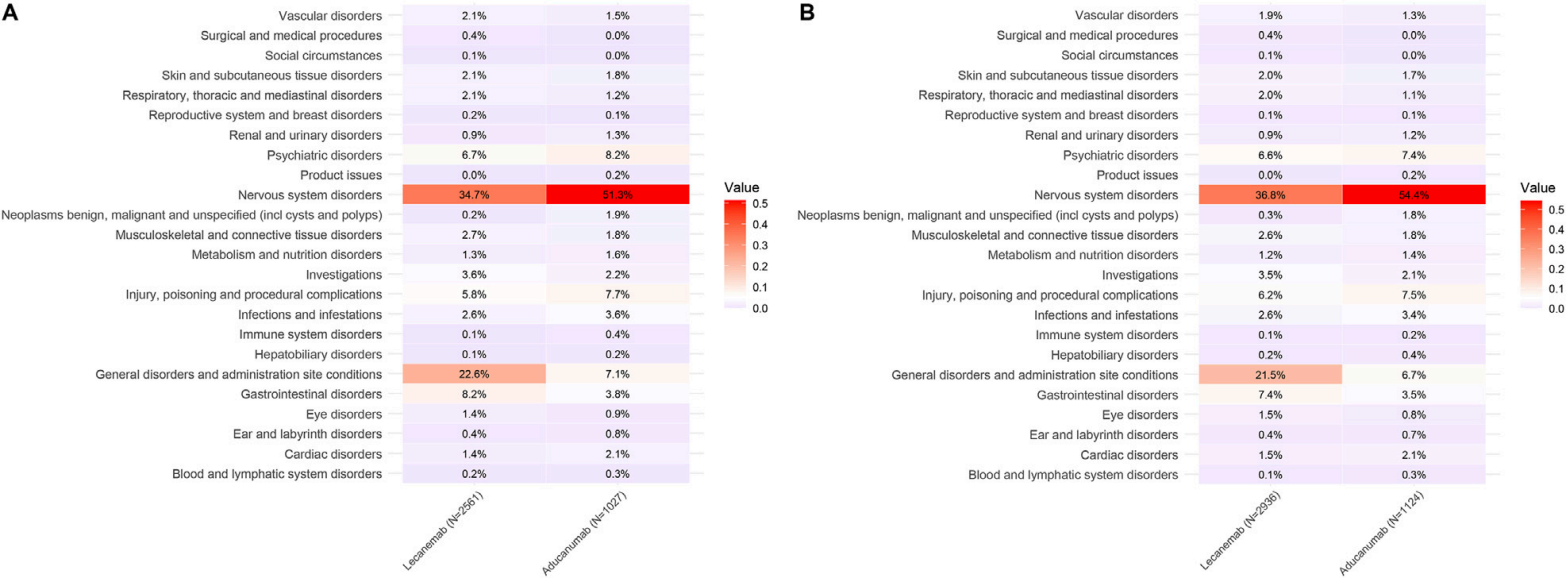
Distribution of SOC for Lecanemab and Aducanumab. Footnotes: **(A)** The distribution of SOC in VigiAccess; **(B)** The distribution of SOC in FAERS; SOC, System Organ Class.

In the VigiAccess database, the top three SOCs for Lecanemab were Nervous system disorders (34.7%), General disorders and administration site conditions (22.6%), and Gastrointestinal disorders (8.2%). Similarly, in the FAERS database, the top three SOCs for Lecanemab were Nervous system disorders (36.8%), General disorders and administration site conditions (21.5%), and Gastrointestinal disorders (7.4%). For Aducanumab, the top three SOCs in both databases were Nervous system disorders, Psychiatric disorders, and Injury, poisoning and procedural complications. Nervous system disorders, general disorders and administration site conditions, as well as gastrointestinal disorders are commonly observed in both Lecanemab and Aducanumab. Specifically, in the VigiAccess database, compared with Aducanumab, the ROR analysis indicates that Lecanemab presents a higher risk in the SOC of General Disorders and Administration Site Conditions (ROR = 3.81, 95% CI = 2.95–4.92) and Gastrointestinal Disorders (ROR = 2.26, 95% CI = 1.60–3.21). In the FAERS database, the risk for General Disorders and Administration Site Conditions (ROR = 3.82, 95% CI = 2.98–4.91) and Gastrointestinal Disorders (ROR = 2.23, 95% CI = 1.58–3.16) is similarly elevated for Lecanemab in comparison to Aducanumab (Table 3). These findings suggest that Aducanumab is associated with more pronounced adverse reactions affecting the central nervous system, whereas Lecanemab poses higher risks in General disorders and administration site conditions and Gastrointestinal disorders. Furthermore, Nervous system disorders emerged as the most prominent SOC for both drugs and the proportion of nervous system-related AEs was significantly higher for Aducanumab than for Lecanemab. The detailed distribution of SOC for Lecanemab and Aducanumab is presented in Figure 3.

**TABLE 3:** Signal detection of Lecanemab and Aducanumab based on ROR analysis.

	Lecanemab	Aducanumab	
SOC	VigiAccess		ROR (95%CI)
Nervous system disorders	889 (34.7%)	527 (51.3%)	0.50 (0.44–0.58)
General disorders and administration site conditions	578 (22.6%)	73 (7.1%)	3.81 (2.95–4.92)
Gastrointestinal disorders	210 (8.2%)	39 (3.8%)	2.26 (1.60–3.21)
	FAERS		
Nervous system disorders	1,080 (36.8%)	612 (54.4%)	0.48 (0.42–0.56)
General disorders and administration site conditions	630 (21.5%)	75 (6.7%)	3.82 (2.98–4.91)
Gastrointestinal disorders	218 (7.4%)	39 (3.5%)	2.23 (1.58–3.16)

SOC, system organ class; ROR, reporting odds ratio.

### Signal detection at the PT level

**FIGURE 4:**
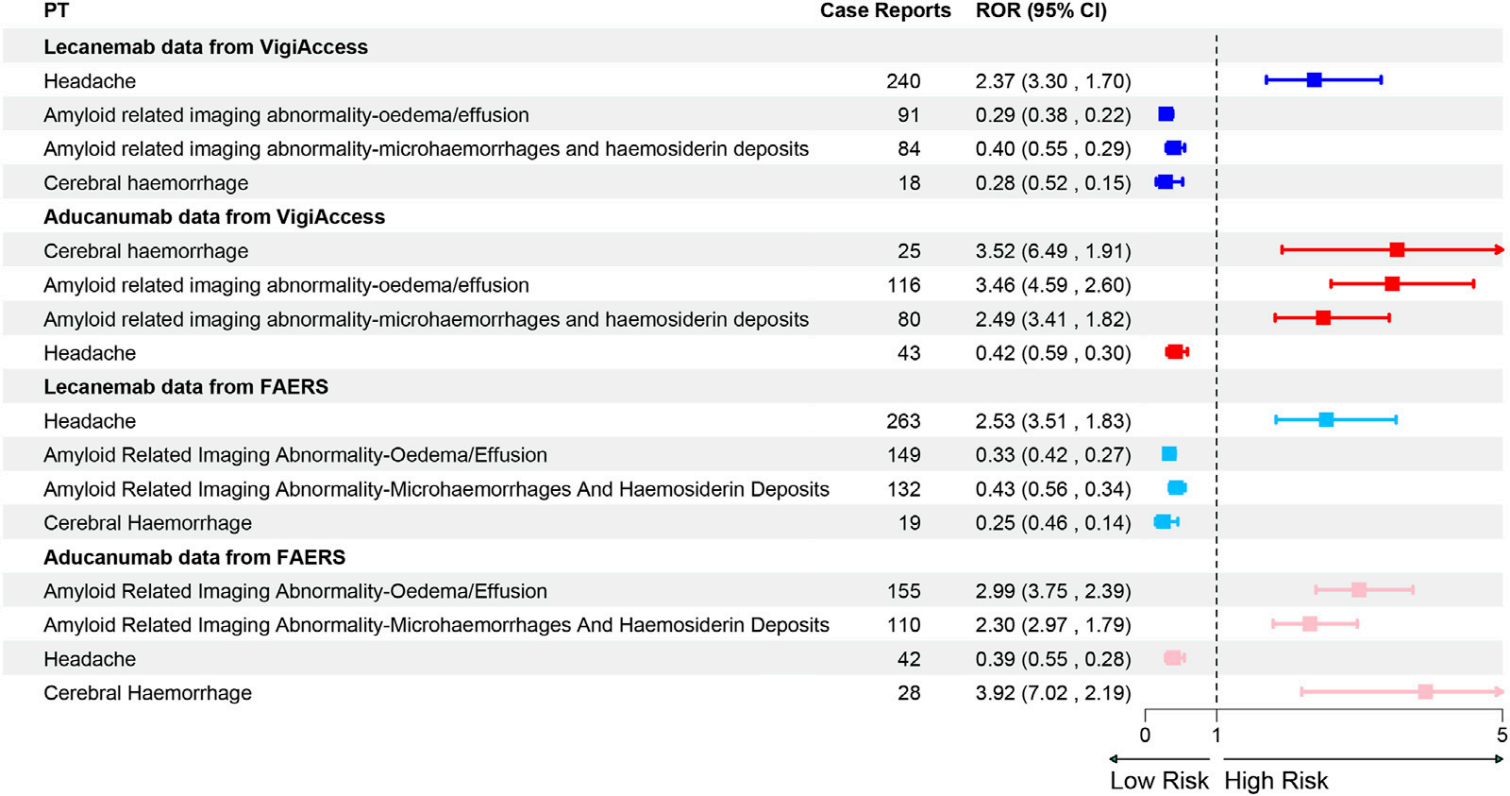
Forest Plot of PT Distribution for Lecanemab and Aducanumab in VigiAccess and FAERS Databases Footnotes: PT, Preferred Term; ROR, Reporting odds ratio.

This study further conducted a comparative analysis of the top 20 PTs for two drugs in two major databases. After excluding possible indications, in the Vigiaccess database, the top 20 PTs for Lecanemab included headache (9.4%), chills (5.0%), fatigue (4.5%), ARIA-oedema/effusion (ARIA-O/E) (3.6%), Infusion related reaction (3.5%), and ARIA-microhaemorrhages and haemosiderin deposits (3.3%); while for Aducanumab, they included ARIA-O/E (11.3%), ARIA-microhaemorrhages and haemosiderin deposits (7.8%), Headache (4.2%), Confusional state (3.4%), Cerebral haemorrhage (2.4%), and Fall (2.2%) (Table 4). In the FAERS database, For Lecanemab, the top 20 PTs included headache (8.96%), ARIA-O/E (5.07%), chills (4.70%), ARIA-microhaemorrhages and haemosiderin deposits (4.50%), fatigue (4.29%), and infusion-related reactions (3.95%). For Aducanumab, the top 20 PTs included ARIA-O/E (13.79%), ARIA-microhaemorrhages and haemosiderin deposits (9.79%), headache (3.74%), confusional state (3.11%), cerebral haemorrhage (2.49%), and fall (2.05%) (Table 5) The ranking of PTs differed slightly between the FAERS and Vigiaccess databases, but the overall reported PTs were similar. It’s worth noting that in the FAERS database, Lecanemab reported Gait disturbance (0.85%), which was not reported in Vigiaccess. The discrepancy between gait disturbance and falls reported in these two databases may be related. Besides, in signal detection at the PT level, the WHO-VigiAccess database shows that Lecanemab is associated with a higher incidence of gastrointestinal (GI) AEs, with the most common GI effects being nausea (3.0%), vomiting (1.9%), and diarrhea (1.2%). In comparison, Aducanumab has a relatively lower incidence of GI effects, with nausea (0.8%) being the most frequently reported. In the FAERS database, Lecanemab also shows higher rates of GI AEs, with nausea (2.86%), vomiting (1.77%), and diarrhea (1.12%) being the most common, whereas Aducanumab has a lower incidence, with nausea (0.71%) being the most common GI event. Therefore, overall, Lecanemab is associated with a higher incidence of GI effects, general disorders, and administration site conditions, while Aducanumab is more predominantly linked to nervous system-related AEs. Overall, AEs associated with Aducanumab were predominantly concentrated in ARIA, particularly ARIA-O/E and ARIA-microhaemorrhages and haemosiderin deposits. In contrast, the most frequently reported AE for Lecanemab was headache. Notably, both ARIA and headache were commonly reported for both drugs. Furthermore, a comparative analysis of the relative ROR between the two drugs revealed that Aducanumab was associated with a significantly higher risk of cerebral haemorrhage and ARIA (ROR >1), while Lecanemab exhibited a higher risk of headache than Aducanumab (Figure 4).

**TABLE 4:** Adverse event distribution of Lecanemab and Aducanumab in the VigiAccess database.

Lecanemab		Aducanumab	
PT	Rate	PT	Rate
Headache	9.4%	Amyloid related imaging abnormality-oedema/effusion	11.3%
Chills	5.0%	Amyloid related imaging abnormality-microhaemorrhages and haemosiderin deposits	7.8%
Fatigue	4.5%	Headache	4.2%
Amyloid related imaging abnormality-oedema/effusion	3.6%	Confusional state	3.4%
Infusion related reaction	3.5%	Cerebral haemorrhage	2.4%
Amyloid related imaging abnormality-microhaemorrhages and haemosiderin deposits	3.3%	Fall	2.2%
Confusional state	3.1%	Seizure	1.9%
Pyrexia	3.1%	Superficial siderosis of central nervous system	1.9%
Nausea	3.0%	Dizziness	1.7%
Dizziness	2.7%	Amyloid related imaging abnormalities	1.5%
Tremor	2.0%	Brain oedema	1.4%
Vomiting	1.9%	Gait disturbance	1.2%
Asthenia	1.3%	Atrial fibrillation	1.1%
Influenza like illness	1.3%	Fatigue	1.0%
Pain	1.3%	Subarachnoid haemorrhage	0.9%
Diarrhoea	1.2%	Urinary tract infection	0.9%
Amyloid related imaging abnormalities	1.2%	Head injury	0.8%
Fall	1.0%	Nausea	0.8%
Feeling cold	0.9%	Cerebrovascular accident	0.7%
Somnolence	0.9%	Cerebral microhaemorrhage	0.7%

PT, preferred term.

**TABLE 5:** Adverse Event distribution of Lecanemab and Aducanumab in the FAERS database.

Lecanemab		Aducanumab	
PT	Rate	PT	Rate
Headache	8.96%	Amyloid related imaging abnormality-oedema/effusion	13.79%
Amyloid related imaging abnormality-oedema/effusion	5.07%	Amyloid related imaging abnormality-microhaemorrhages and haemosiderin deposits	9.79%
Chills	4.70%	Headache	3.74%
Amyloid related imaging abnormality-microhaemorrhages and haemosiderin deposits	4.50%	Confusional state	3.11%
Fatigue	4.29%	Cerebral haemorrhage	2.49%
Infusion related reaction	3.95%	Fall	2.05%
Confusional state	2.96%	Superficial siderosis of central nervous system	2.05%
Pyrexia	2.90%	Seizure	1.78%
Nausea	2.86%	Dizziness	1.51%
Dizziness	2.49%	Amyloid related imaging abnormalities	1.51%
Vomiting	1.77%	Brain oedema	1.25%
Tremor	1.74%	Fatigue	0.98%
Amyloid related imaging abnormalities	1.46%	Gait disturbance	0.98%
Asthenia	1.26%	Atrial fibrillation	0.98%
Pain	1.23%	Urinary tract infection	0.80%
Diarrhoea	1.12%	Subarachnoid haemorrhage	0.80%
Somnolence	0.99%	Head injury	0.71%
Fall	0.95%	Nausea	0.71%
Feeling cold	0.89%	Cerebrovascular accident	0.62%
Gait disturbance	0.85%	Cerebral microhaemorrhage	0.62%

PT, preferred term.

### Logistic regression analysis

Nervous system disorders were the most prominent SOC for both Lecanemab and Aducanumab. After excluding reports with missing data, a total of 1,321 cases associated with Lecanemab and Aducanumab, which included information on age, sex, and weight, were retained. In the univariate analysis, our results indicated that Aducanumab was associated with a higher risk of nervous system disorders compared to Lecanemab (OR = 4.58, 95% CI: 3.43–6.19, P < 0.001). The results of the multivariate logistic regression analysis were consistent (OR = 4.72, 95% CI: 3.53–6.39, P < 0.001). Both univariate and multivariate logistic regression analyses revealed no significant association between age, sex, and the occurrence of nervous system disorders (Table 6).

**TABLE 6:** Univariate and multivariate logistic regression analysis of risk factors for nervous system disorders.

Group	Nervous system disorders	No-nervous system disorders	OR (univariable)	OR (multivariable)
Age				
<45	4 (66.7%)	2 (33.3%)	Reference	Reference
≥45, <65	76 (48.4%)	81 (51.6%)	2.13 (0.40–15.69, P = 0.390)	1.65 (0.31–12.15, P = 0.573)
≥65, <74	224 (45.2%)	272 (54.8%)	2.43 (0.47–17.63, P = 0.308)	1.73 (0.33–12.58, P = 0.531)
≥75	346 (52.3%)	316 (47.7%)	1.83 (0.35–13.24, P = 0.488)	1.25 (0.24–9.09, P = 0.797)
Sex				
Female	362 (47.3%)	403 (52.7%)	Reference	Reference
Male	288 (51.8%)	268 (48.2%)	0.84 (0.67–1.04, P = 0.108)	0.80 (0.64–1.01, P = 0.065)
Drug				
Lecanemab	580 (57.3%)	432 (42.7%)	Reference	Reference
Aducanumab	70 (22.7%)	239 (77.3%)	4.58 (3.43–6.19, P < 0.001)	4.72 (3.53–6.39, P < 0.001)

### Serious adverse events for two drugs

**FIGURE 5:**
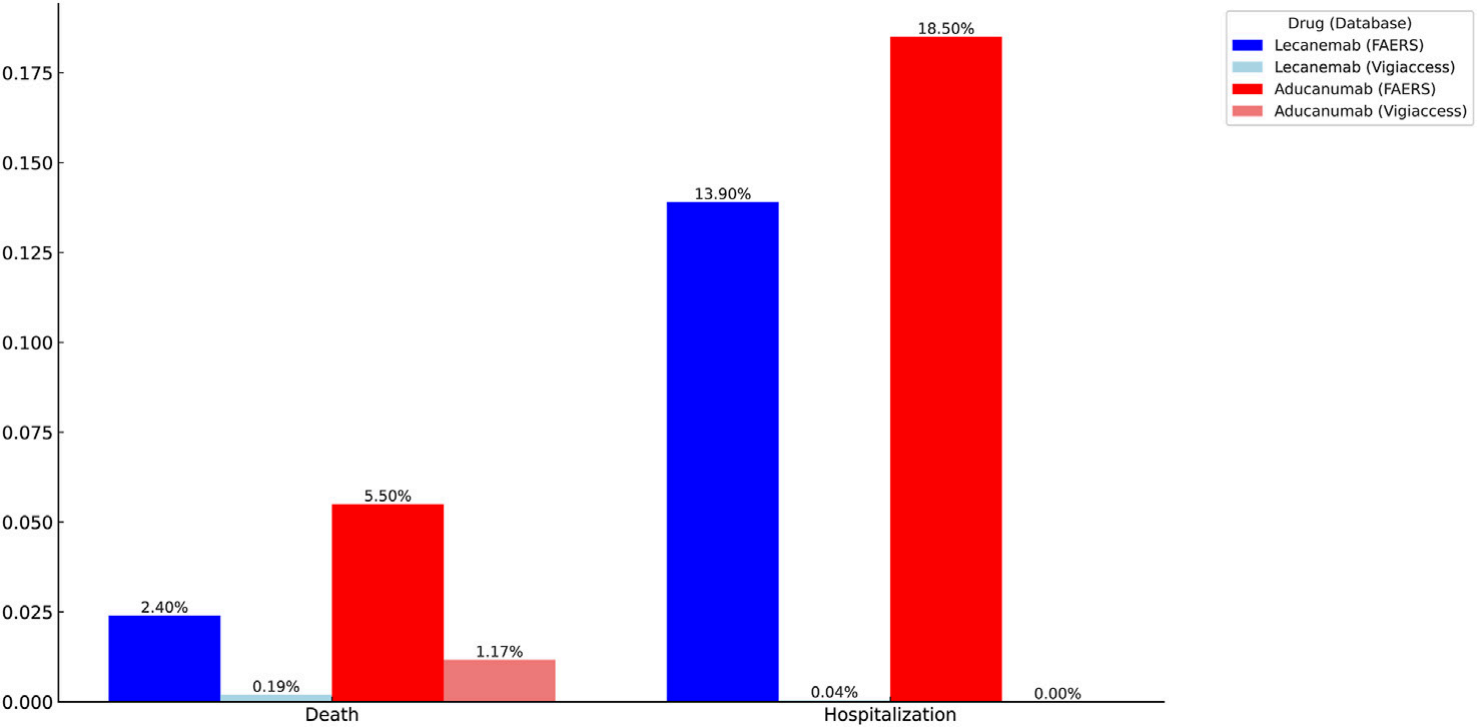
Identification of serious adverse events for Lecanemab and Aducanumab using VigiAccess and FAERS databases.

In an analysis of two related serious AEs, we observed higher rates of hospitalization (18.50%) and mortality (5.50%) for Aducanumab than for Lecanemab (13.90%) and mortality (2.40%) in the FAERS database. In the Vigiaccess database, we found that Aducanumab (1.17%) had a significantly higher mortality rate than Lecanemab (0.19%). Therefore, Lecanemab may be a better choice than Aducanumab (Figure 5).

### Similarities and differences in ADRs between the two drugs

Finally, this study analyzed and organized the similarities and differences in ADRs of two drugs in the VigiAccess and FAERS databases. Detailed data was provided in the supplementary material. In the blood and lymphatic systems, Aducanumab showed a wider range of adverse reactions compared to Lecanemab, with anemia being a common AE for both. Thrombocytopenia is more common in Lecanemab, while the more common PT in Aducanumab is Platelet dysfunction and Myelosuppression. In respiratory disorders, epistaxis and rhinorrhoea were shared AEs. Regarding cardiac conditions, both medications may induce atrial fibrillation. These AEs are infrequently mentioned in the medication guides but remain noteworthy. Overall, Lecanemab demonstrated a higher incidence of general disorders and administration site conditions compared to Aducanumab. The most prevalent SOC for both drugs was nervous system disorders, with the highest number of reported cases. Additionally, Aducanumab had a significantly higher incidence of cerebral events, such as ARIA and cerebral hemorrhage, compared to Lecanemab.

## Discussion

This study provides valuable and precise insights into the safety profiles of Lecanemab and Aducanumab in real-world clinical settings by analyzing ADRs associated with these medications from the VigiAccess and FAERS databases. The findings indicate that the majority of patients are aged 65 and above, reflecting the current demographic concentration of AD predominantly in this age group (Hou et al., 2019). Geographically, the sample sizes for both Lecanemab and Aducanumab are primarily from the United States, correlating with the market availability of these drugs in the U.S.

Evaluating drug safety is central to new drug development and clinical application, particularly for novel therapeutics such as Lecanemab and Aducanumab. A study involving early AD patients revealed that those receiving anti-Aβ therapy experienced a slower decline in cognitive abilities, highlighting the efficacy of the treatment; however, AEs warrant further attention (McDade et al., 2022; van Dyck et al., 2023). Real-world data, especially analyses based on spontaneous reporting systems (SRS), can complement clinical trials by monitoring ADRs from a large user base (Noguchi et al., 2019). This study, through a combined analysis of VigiAccess and FAERS, elucidates the distribution characteristics of ADRs within the SOC and differences in PT for the two drugs. The study found that the most common SOCs for Lecanemab are Nervous System Disorders, General Disorders and Administration Site Conditions, and Gastrointestinal Disorders, with the most frequent AEs being headache, ARIA, chills, and fatigue. For Aducanumab, the predominant SOCs are Nervous System Disorders, Psychiatric Disorders, and Injury, Poisoning, and Procedural Complications, with ARIA being the most common AE. Further ROR analysis indicated that Aducanumab carries a significantly higher risk of ARIA and cerebral hemorrhage compared to Lecanemab, while Lecanemab is associated with a higher risk of headache. Additionally, Aducanumab was linked to higher hospitalization and mortality rates. The study also compared the similarities and differences between the two drugs, underscoring the significance of these findings.

Lecanemab is a monoclonal IgG1 antibody with a high affinity for binding protofibrils of Aβ protein. Its efficacy was convincingly demonstrated in murine experiments (Söderberg et al., 2023). Furthermore, Lecanemab showed significant plaque-clearing capability and therapeutic effects in Phase 2b clinical trials (Swanson et al., 2021). However, the risk-benefit profile of Lecanemab remains contentious. Current studies and meta-analyses indicate that Lecanemab increases the risk of ARIA-E and ARIA-H, and anticoagulant therapy further elevates the risk of bleeding (Cummings et al., 2023; Abdelazim et al., 2024). Atwood et al. also argue that the risks of Lecanemab outweigh its benefits (Atwood and Perry, 2023). Conversely, some studies suggest that ARIA is typically transient and asymptomatic, often occurring early in treatment with a reduced risk in later stages (Barakos et al., 2022). Our research indicates that Nervous System Disorders and General Disorders and Administration Site Conditions are the most common SOCs for Lecanemab, with headache, ARIA, chills, and fatigue being the most frequently reported PTs. Therefore, the occurrence of ARIA necessitates cautious continuation of treatment and regular monitoring. Headache, chills, and fatigue, which have not been sufficiently emphasized in previous studies, should also receive increased attention.

Clarity AD was an 18-month treatment (Core study), multicenter, double-blind, placebo-controlled, parallel-group study with open-label extension (OLE) in participants with early AD. In the Clarity AD trial, the most frequently reported adverse events in the Lecanemab group (>10%) were infusion-related reactions (24.5%). The incidence of amyloid-related imaging abnormalities with edema or effusion (ARIA-E) was 12.6% among Lecanemab-treated patients, significantly higher than in the placebo group. Most ARIA-E cases were mild to moderate (91%), asymptomatic (78%), occurred within the first 3 months of treatment (71%), and resolved within 4 months of detection (81%). Symptomatic ARIA-E developed in 2.8% of participants, with common symptoms including headache, visual impairment, and confusion (van Dyck et al., 2023; Honig et al., 2024). During the OLE phase, nine deaths were recorded, four of which were deemed likely related to the study treatment. Among the 24 deaths in the Core + OLE groups, three were attributed to intracerebral hemorrhage (ICH) (Honig et al., 2024). Additionally, the incidence of ARIA in Asian participants was notably lower compared to other regions in this global study. Only 10 out of 153 Asian participants experienced ARIA, representing a rate of 6.5%. For Japanese participants specifically, the rate was even lower at 4.5% among 88 individuals. Clinical observations align with these findings, as Iwatsubo reports that approximately 5% of patients develop ARIA-E or ARIA-H (Author Anonymous, 2025).

A retrospective observational study used administrative claims data from the Komodo Research Database Of the 3,155 patients treated with Lecanemab in the U.S., 4% were concurrently using anticoagulants, which are recognized as a risk factor for more severe adverse effects. A study from Columbia University reported 148 patients treated with Lecanemab. Overall, 9% of patients interrupted or stopped treatment, 6% due to ARIA, 1% due to persistent infusion reactions, and 2% due to patients' unwillingness to continue treatment. During treatment, 14% of patients experienced an infusion reaction (headache, chills, fatigue), predominantly after the first three infusions but not exclusively so. The incidence of ARIA-E is 6%. One patient developed severe ARIA, leading to aphasia shortly after the third infusion. Despite prompt diagnosis via MRI, the patient subsequently died following refractory focal status epilepticus. This was the only reported death (Author Anonymous, 2025). According to neurologist Shawn Kile, 234 patients were treated with Lecanemab. So far, 15 patients have been infected with ARIA-E, three of whom were severe on MRI, causing confusion and headaches. One man had a lasting neurological defect and lost a quarter of his vision. In addition, three patients experienced major bleeding 1–3 months after ARIA withdrawal from Lecanemab, suggesting that side effects may be delayed. However, there have been no deaths associated with Lecanemab. Again, Joy snyder reports on the latest situation. Similar to those in the trial, 13% developed ARIA-E, two-thirds of which were mild and asymptomatic. Seven people developed symptoms, two of them severe enough to require hospitalization. It is worth noting that most ARIA occurs around the time of the fifth infusion. Due to British restrictions on Lecanemab use for non-homozygotes, Richard Perry excluded this group, leaving 1,521 participants without E4 homozygotes. The results found that the incidence of ARIA-E decreased from 13% to 9%, ARIA-H from 17% to 13%, microbleeds from 14% to 10%, and superficial siderosis from 6% to 4% (Author Anonymous, 2025).

Aducanumab, developed by Biogen, is a recombinant human antibody that binds to amino acids 3-7 of the Aβ peptide. After initial Phase 3 trial analyses failed to meet primary endpoints, the development of Aducanumab was temporarily halted in 2019. Subsequent data reanalysis demonstrated significant plaque-clearing capability and therapeutic signals, leading to FDA approval in 2021 as the first anti-Aβ therapy for AD (Sevigny et al., 2016; Arndt et al., 2018; Tolar et al., 2020; Tolar et al., 2021). ARIA is the primary AE associated with this medication. Recent trials and meta-analyses indicate that ARIA is more prevalent in carriers of the apolipoprotein E ε4 (ApoE ε4) allele, encompassing both ARIA-H and ARIA-E. Additionally, Aducanumab increases the risk of cerebrovascular events, such as microhemorrhages and edema (Loomis et al., 2024; Arroyo-Pacheco et al., 2025). This is attributed to the association of ApoE-ε4 with Aβ accumulation in cerebral amyloid angiopathy (CAA), which compromises the integrity of neurovascular units, leading to immune dysregulation and the induction of ARIA and microhemorrhages (Cheng et al., 2014; Foley and Wilcock, 2024). In two Phase 3 clinical trial of Aducanumab, ARIA occurred in about 40 percent of participants. This trial revealed that 425 out of 1,029 patients (41.3%) developed ARIA. ARIA-E occurred in 362 patients (35.2%), and 94 patients (26.0%) developed ARIA-related symptoms, including headaches, confusion, dizziness, and nausea (Salloway et al., 2022). Aducanumab reports showed 128 peaks in 2022 (44.1%) and 2023 (39.0%), and Aducanumab was discontinued between February and May 2024 (Alzheimer’s Association, 2024). In addition, Health Canada also believes that based on the evidence in 2021, Aducanumab is not ripe for approval. The Japanese Ministry of Health also refused to approve Aducanumab for a Phase I trial in Japan (Thussu et al., 2024).

This study found that Aducanumab poses a higher risk of ARIA and cerebrovascular events compared to Lecanemab. An experiment isolating therapeutic Aβ antibodies from human leptomeningeal tissue demonstrated that Lecanemab has a lower binding rate to CAA protofibrils, consistent with its relatively lower incidence of ARIA-E (12.6%), whereas Aducanumab exhibits a higher binding rate and a significantly increased occurrence of ARIA-E (25%–35%) (Söderberg et al., 2024). A subgroup analysis from Japan on various Aβ antibody medications revealed the highest incidence of ARIA-E in the high-dose Aducanumab group (Toda et al., 2024). Joel et al. reported a case where Aducanumab treatment led to refractory status epilepticus associated with severe ARIA (Neves Briard et al., 2024). Consequently, Aducanumab is generally considered to offer only moderate benefits for AD. Its high cost and elevated ARIA event rate have sparked debates on whether its benefits outweigh the risks and financial burdens on the healthcare system (Vaz et al., 2022). Currently, professional clinicians and Walsh et al. argue that accelerated approval is inappropriate until the risk-benefit profile of Aducanumab is fully clarified (Walsh et al., 2021; Dhruva et al., 2023). Rahman et al. also recommend that future research should focus on addressing the efficacy and safety concerns of Aducanumab to confirm its risk-benefit status (Rahman et al., 2023). This study observed that, compared to Aducanumab, Lecanemab has a lower incidence of ARIA and a relatively smaller risk of cerebral hemorrhage. For patients with high risks of cerebrovascular or other neurological diseases, Lecanemab may be the safer option. Additionally, the mortality risk associated with Aducanumab is significantly higher than that of Lecanemab, suggesting that clinicians should exercise greater caution and closely monitor patient responses when using Aducanumab. Moreover, this study identified less frequently reported AEs, including central nervous system surface iron deposition, states of confusion, and epileptic seizures, which are more common with Aducanumab use. Furthermore, this study identified new SOCs and AEs not previously reported in the drug labels and conducted comparative analyses of the similarities and differences between the two medications. The identification of these new AEs requires meticulous attention and further investigation. In clinical practice, it is recommended that healthcare providers closely monitor potential new ADRs and adopt personalized treatment strategies based on the distinct ADR profiles of Lecanemab and Aducanumab, considering individual patient characteristics.

In conclusion, although Lecanemab and Aducanumab have demonstrated certain efficacy in treating the etiology of AD and offer new hope to AD patients, both this study and the drug labels indicate that Nervous System Disorders are the most common SOCs, and both medications are susceptible to ARIA. Notably, Aducanumab’s higher risk of ARIA and cerebrovascular events necessitates more cautious application. Overall, through an in-depth analysis of the ADRs associated with Lecanemab and Aducanumab, clinicians have gained a clearer understanding of the frequency and nature of AEs observed in a real-world. This analysis has facilitated the development of effective strategies for monitoring and managing these AEs. Furthermore, it has prompted discussions on the cost-effectiveness and risk-benefit profiles of treating AD. Additionally, examining the ADRs of Lecanemab and Aducanumab has highlighted the importance of obtaining informed consent from patients with early-stage AD, ensuring they fully comprehend both the potential risks and the limited benefits of treatment. Although Biogen has decided to discontinue further studies of Aducanumab, its development has paved the way for new research directions in monoclonal antibodies targeting Aβ and tau proteins. Our research underscores the critical need to prioritize ADR evaluation in future drug development processes. Future research should prioritize the assessment of long-term safety and risk-benefit evaluations, and through the refinement of treatment strategies and drug design, provide safer and more effective therapeutic options for AD patients.

Despite evaluating the ADRs of Lecanemab and Aducanumab from multiple dimensions, this study acknowledges certain limitations. Firstly, both WHO-VigiAccess and FAERS databases rely on voluntary reporting, meaning not all AEs are reported. This could underestimate the true incidence of ADRs. Secondly, certain ADRs may be overreported due to heightened media attention, regulatory warnings, or clinical awareness, skewing results. Additionally, different countries have different pharmacovigilance systems, which may lead to variation in AE reporting rates. Moreover, the databases do not always include detailed dosage, treatment duration, or drug compliance data, limiting deeper analysis of risk factors. Finally, since the study is observational and retrospective, it cannot control for potential biases inherent in real-world data collection. Future research should prioritize cohort studies or randomized controlled trials (RCTs) to validate the current findings and assess the relative efficacy and safety of Lecanemab and Aducanumab across diverse patient populations. Additionally, it is imperative to investigate the underlying mechanisms of any ADRs that may occur.

## Conclusion

This study focused on analyzing the ADRs of Lecanemab and Aducanumab and compared the differences between the two drugs. The results showed that ARIA was the most common AE for both drugs, but Lecanemab had a lower risk of ARIA and cerebral hemorrhage compared to Aducanumab. Therefore, Lecanemab may be a more preferred treatment option. These results underscore the necessity of conducting further clinical practice research to attain a clearer understanding of the long-term safety and efficacy of both drugs.

## Data Availability

The datasets presented in this study can be found in online repositories. The names of the repository/repositories and accession number(s) can be found in the article/Supplementary Material.
